# Clinical Performance of Posterior Tooth‐Supported Cantilevered Prostheses With a Follow‐Up of ≥ 3 Years: A Systematic Review and Meta‐Analysis

**DOI:** 10.1155/ijod/9315051

**Published:** 2026-09-30

**Authors:** Pardis Ghorbani, Momen A. Atieh, Haitham Elbishari, Nabeel H. M. Alsabeeha, Fatemeh Amir-Rad

**Affiliations:** ^1^ Hamdan Bin Mohammed College of Dental Medicine Mohammed Bin Rashid University of Medicine and Health Sciences Dubai Health Dubai UAE mbruniversity.ac.ae; ^2^ Sir John Walsh Research Institute Faculty of Dentistry University of Otago Dunedin New Zealand otago.ac.nz; ^3^ School of Dentistry University of Jordan Amman Jordan ju.edu.jo; ^4^ School of Medicine, Dentistry and Biomedical Sciences Queen’s University Belfast UK qub.ac.uk; ^5^ The Faculty of Biology, Medicine and Health School of Medical Science Division of Dentistry University of Manchester Manchester England UK manchester.ac.uk; ^6^ Department of Dental Services Emirates Health Services Ras Al-Khaimah UAE

**Keywords:** cantilevered prosthesis, clinical performance, fixed dental prosthesis, meta-analysis, survival, systematic review, tooth-supported prosthesis

## Abstract

**Objectives:**

This study aimed to assess the survival rate and complications of posterior tooth‐supported cantilevered fixed dental prostheses (C‐FDPs) over a minimum 3‐year observation period.

**Materials and Methods:**

The systematic review followed Preferred Reporting Items for Systematic Reviews and Meta‐Analyses (PRISMA) guidelines. Relevant studies were identified by electronic searches in PubMed, Scopus, Cochrane, and Embase databases. Two reviewers selected studies on posterior tooth‐supported C‐FDPs applying the inclusion criteria, extracted data, and assessed quality using the Joanna Briggs Institute (JBI) tool. Meta‐analyses focused on the survival and failure rates of C‐FDPs.

**Results:**

Ten studies were included. The survival rates of the C‐FDPs were 95.57% over 3 years and 93.45% over 5 years. Premolar‐ and molar‐replacements showed no statistically significant differences in 3 and 5 years (96.24% and 93.82% for premolar‐ versus 94.49% and 90.98% for molar‐replacing prostheses). Maxillary and mandibular replacements had similar survival rates of 96.29% and 93.89% in 3 years and 5 years, respectively. Mesial‐ and distal‐cantilevered prostheses had survival rates of 100% at both time points.

**Conclusion:**

Tooth‐supported C‐FDPs exhibit comparable performance in the maxilla versus the mandible, premolars versus molars, and mesial versus distal abutments, with minimal reported biological and mechanical complications. However, the limited number of studies underscores the need for further high‐quality clinical research to validate these results.

## 1. Introduction

Despite significant advancements in dentistry and improvements in global oral health [1], tooth loss remains a leading problem across various populations and can adversely affect the oral health‐related quality of life. Tooth loss can result from various causes, including dental caries, periodontal disease, trauma, and congenital conditions, with dental caries being the leading cause of tooth loss worldwide [2, 3]. Moreover, a positive association between higher incidence of tooth loss due to caries and greater indices of decayed, missed, and filled teeth (DMFT) has been demonstrated among different populations [4–7].

Single tooth loss is one of the most common yet challenging clinical conditions as it can significantly impact the overall treatment plan for oral rehabilitation. Following tooth loss, rotation or drifting of adjacent teeth and overeruption of the opposing dentition with subsequent occlusal alteration are not uncommon [8–11]. Most of these complications, however, have been observed to occur in the first 5 years following tooth loss, particularly in younger patients [11].

Treatment options for the replacement of single missing teeth generally include the use of fixed or removable prostheses. Although removable prostheses are less invasive and more affordable, patient acceptance and compliance is very challenging [12]. Implant‐supported single crown prostheses, on the other hand, have shown long‐term survival and predictability [13–15]. However, financial, anatomical, and other patient‐related constraints can limit their application. Fixed–fixed tooth‐supported prostheses, while a standard treatment option for decades, are, nevertheless, destructive when sound or minimally restored abutment teeth are involved. Loss of pulp vitality in 11.3% [16] and pulpal necrosis in 15.6% and 32.5% of abutment teeth prepared for single crowns and fixed dental prostheses (FDPs), respectively, has been reported [17]. These biological risks are closely linked to the extent of tooth reduction required for retainer preparation. Conventional full‐coverage retainers necessitate circumferential axial and occlusal reduction, whereas resin‐bonded FDPs (RBFDPs) (also known as resin‐bonded bridges [RBBs]) retainers require only minimal, enamel‐confined preparation, thereby preserving substantially more sound tooth structure [18, 19]. Finite element analyses further suggest that RBFDP designs shift functional stress toward the adhesive bonding interface rather than the abutment tooth itself, in contrast to the more uniform intracoronal stress transfer seen with conventionally prepared abutments [20]. Alternatively, the tooth‐supported cantilevered FDP (C‐FDP) can be a more conservative treatment option compared to the fixed–fixed prostheses. A C‐FDP is a prosthesis that restores one or more dental units and is retained to an adjacent abutment on one side [21]. The retainer of a C‐FPD can be a ceramic or metal wing bonded to a minimally prepared abutment tooth, an inlay or onlay prosthesis, or a partial or full coverage crown. A survival rate between 87.7% and 100% has been reported for C‐FPDs of various designs with a follow‐up period between 3 and 5 years [22–24].

Survival outcomes of anterior tooth‐supported resin‐bonded C‐FDPs have been well reported in the literature [25–27]. On the other hand, survival outcomes of tooth‐supported C‐FDP replacing a single missing posterior tooth remain scarce and inconclusive. Therefore, the aim of this systematic review was to evaluate survival outcomes and incidence of biological and mechanical complications of tooth‐supported C‐FDP replacing one missing posterior tooth with a mean observation period of at least 3 years.

## 2. Material and Methods

The present systematic review was developed following the principles of Preferred Reporting Items for Systematic Reviews and Meta‐Analyses (PRISMA 2020). The eligibility criteria for the review were defined using the population, intervention, comparison, and outcome (PICO) framework [28, 29]:

Population: Human adults of any gender with a single missing premolar or molar tooth who require replacement with tooth‐supported C‐FDPs.
Intervention: Single tooth‐supported C‐FDPs using a single retainer of any design—conventional or resin‐bonded—and material.
Comparison: Clinical outcomes of premolar‐ versus molar, maxillary‐ *versus* mandibular, and mesial‐ versus distal tooth‐supported C‐FDPs.
Outcomes: survival rate and incidence of biological and mechanical complications.

The study was registered with the National Institute for Health Research (NIHR) under PROSPERO ID CRD42023416958. Ethical approval was not required for this systematic review.

### 2.1. Inclusion Criteria

This review included randomized, nonrandomized, observational, and cohort clinical studies that evaluated posterior tooth‐supported C‐FDPs of different designs—conventional or resin‐bonded—and materials for single tooth replacement, with a minimum observation period of 3 years.

### 2.2. Exclusion Criteria

In vitro studies, experimental and histological studies, and clinical studies that lacked sufficient data, where attempts to obtain more details from the authors were unsuccessful, were excluded. In addition, studies involving more than one abutment tooth and studies replacing more than one missing tooth were excluded.

### 2.3. Outcome Measures

Primary outcome: survival rate of prostheses.

Secondary outcomes: incidence of biological and mechanical complications.

### 2.4. Search Strategy

An electronic search was conducted to identify all relevant articles published in the English language up to the end of June 2026. The following databases were searched: PubMed (Medline), Scopus, Cochrane, and Embase. The search was complemented by a manual search through the reference lists of the selected papers. Two reviewers (Pardis Ghorbani and Fatemeh Amir‐Rad) performed the search independently and in duplicate using defined search terms in each electronic database (Supporting Information: Appendix A). The following keywords were used for the manual search: posterior cantilever, cantilever, cantilevered restoration, RBB, RBFDP, FDP, bridge, denture, adhesive bridge, Maryland bridge, dental restoration, dental restoration failure, prosthesis failure, and treatment outcome.

### 2.5. Case Definition

Survival was defined as the prostheses remaining in place with or without any alterations [30]. Mechanical complications included fracture or chipping of the prostheses or the abutment tooth and debonding of the C‐FDP. Biological complications included developing secondary caries on the abutment tooth, periodontal attachment loss, and a subsequent increase in probing depth.

### 2.6. Study Selection

The titles and abstracts of retrieved citations were screened for eligibility by two independent reviewers (Pardis Ghorbani and Fatemeh Amir‐Rad). After the exclusion of irrelevant papers, the full texts of potential studies were examined based on the inclusion and exclusion criteria set for the review. Any disagreement between reviewers was resolved by discussion to reach consensus or consultation with a third reviewer (Haitham Elbishari). In studies where the individual prostheses had varying times in service, only prostheses with at least 3 years in function were included. In order to keep a record of all studies retrieved from each database, Rayyan, a web‐based automated screening tool for systematic review, was utilized [31].

### 2.7. Quality Assessment

The quality of the included studies was evaluated using the Joanna Briggs Institute (JBI) assessment tool for case series studies [32]. This tool consists of 10 questions that appraise various aspects of study quality. For example, questions 1–5 assess the process and clarity of participant inclusion, while questions 6 and 7 focus on the reporting of patient demographics and clinical information. Questions 8 and 9 evaluate the clarity of outcome reporting and the demographic data of the study sites, respectively, while question 10 assesses the appropriateness of the statistical analysis of the study results. Each question could be answered with “yes,” “no”, “unclear,” or “not applicable.” A “yes” response was assigned a score of + 1, with a total score for each study ranging from 0 to 10. Studies were rated as low quality if they scored between 0 and 3, medium quality if they scored between 4 and 6, and high quality if they scored 7 or more.

### 2.8. Data Collection

Using a data extraction form, two reviewers (Pardis Ghorbani and Fatemeh Amir‐Rad) independently collected the following information from each included study: title, study type, year, authors, setting (university/hospital/private practice), sample size, retainer design, abutment tooth preparation, site of the cantilever (mesial or distal and maxilla or mandible), prosthetic material, location of the missing tooth (premolar or molar), vitality status of the abutment tooth, type of cement, and observation period. Data visualization and infographic generation were assisted by NotebookLM (Google, Mountain View, CA, USA) [33].

### 2.9. Outcome Measures

The primary outcome of the study was survival rate, while the secondary outcomes were biological and/or mechanical complication rates of posterior C‐FDPs after a defined period of time, which were expressed in percentages. The outcomes were compared by subgrouping into premolar versus molar C‐FDPs, maxillary versus mandibular C‐FDPs, and mesial versus distal C‐FDPs.

### 2.10. Data Synthesis

Annual failure rates were calculated by dividing the total number of failures by the corresponding total prosthesis years of follow‐up and were expressed per 100 prosthesis years. Estimated 3‐ and 5‐year survival rates were derived from the annual failure rate assuming a constant failure rate over time using the exponential survival function S(t) = exp(−rt), where *r* represents the annual failure rate and t the follow‐up time in years. For groups in which more than one study contributed data, study‐specific incidence rates were pooled using an inverse‐variance random‐effects model on the logarithmic scale and subsequently back‐transformed to obtain summary incidence rates and 95% confidence intervals. A continuity correction of 0.5 events was applied to studies reporting zero failures to permit estimation on the logarithmic scale. The between‐study variance was estimated using the DerSimonian–Laird method. Comparative analyses of failure according to prosthesis location (premolar versus molar and maxilla versus mandible) were expressed as risk ratios (RRs) with 95% confidence intervals and pooled using a random‐effects model in Review Manager (RevMan), Version 5.4 (The Nordic Cochrane Centre, The Cochrane Collaboration, Copenhagen, Denmark).

## 3. Results

### 3.1. Search and Included Studies

A total of 29,031 citations were identified from all searches, and 29,002 were excluded after title and abstract screening and the removal of duplicate studies. After full‐text assessment of the remaining 29 studies, 19 were excluded, with the excluded studies and reasons for exclusion listed in Supporting Information: Appendix B. The reasons for exclusion included unreported mean observation time, insufficiently reported details, double‐retainer cantilever RBFDPs designs, observation periods shorter than 36 months, duplicated samples, and cantilevered prostheses involving more than one abutment tooth. Ten studies were finally included in this systematic review [22, 24, 34–38] (Figure 1). The agreement between the two reviewers was calculated using Kappa statistics, which revealed a substantial agreement (0.8).

**Figure 1:**
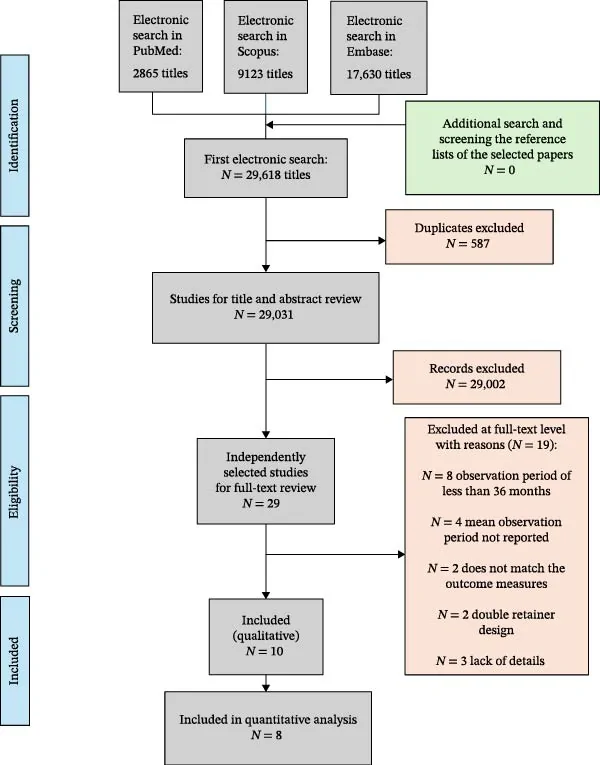
Search strategy.

### 3.2. Characteristics of the Studies

The characteristics of the included studies are summarized in Table 1. Ten studies, published between 1996 and 2025, evaluated 380 posterior C‐FDPs [22, 24, 34–41]. Five were retrospective [22, 24, 34, 39, 40], four were prospective [35, 36, 38, 41], and one was an observational cohort study [37]. Most prostheses replaced premolars (319/380), and the reported follow‐up ranged from 36.0 to 115.2 months. Arch location was available for 343 prostheses, including 181 maxillary and 162 mandibular restorations [22, 34–39, 41]. The cantilever direction was reported for 87 prostheses and was predominantly mesial (75/87) [24, 35–37, 41].

**Table 1:** Study‐level characteristics of the included studies.

Study	Study design	Setting	Premolar/molar C‐FDPs (*n*)	Maxillary/mandibular C‐FDPs (*n*)	Cantilever direction mesial/distal (*n*)	Abutment tooth preparation category	Retainer configuration	Material	Vitality of abutment tooth	Type of cement	Observation period (months)
Hussey and Linden [36]	Prospective clinical trial	University	Premolar (10) molar (1)	Maxilla (8) mandible (3)	Mesial (6) distal (5)	Enamel‐confined/minimal	Palatal/lingual wing with wraparound design and cingulum rest	Porcelain fused to metal	Vital	Resin adhesive^a^	36.8
Olsson et al. [37]	Observational cohort study	General dental practice	Premolar (6) molar (1)	Maxilla (5) mandible (2)	Mesial (2) distal (5)	Full‐crown	Full‐crown retainer with deep chamfer margins	In‐Ceram Alumina	NR	Zinc phosphate^b^	76.0
Botelho et al. [39]	Retrospective clinical study	Dental hospital	Premolar (91) molar (19)	Maxilla (68) mandible (42)	NR	Minimally invasive preparation confined to enamel	C‐shaped retainer strengthened to a D‐shaped retainer with occlusal bar	Cast nickel–chromium alloy	Vital; 7 abutments endodontically treated	Resin adhesive^a^	51.7
Lam et al. [40]	Retrospective case–control study	Dental hospital	Premolar (13) molar (9)	NR	NR	Minimally invasive preparation	D‐shaped retainer with occlusal bar	Cast nickel–chromium alloy	NR	Resin adhesive	115.2
Sailer et al. [24]	Retrospective clinical study	Private practice	Premolar (14) molar (1)	NR	Mesial (15) distal (0)	Defect‐oriented tooth preparation	Inlay, onlay, wing, partial crown, or combination, selected according to existing defects	Glass ceramic^f^	Vital	Resin adhesive^c^	72.0
Botelho et al. [22]	Retrospective clinical study	Dental hospital	Premolar (83) molar (17)	Maxilla (60) mandible (40)	NR	Minimally invasive preparation	D‐shaped retainer with occlusal bar	Porcelain fused to metal	Vital initially, some teeth underwent endodontic treatment	Resin adhesive^a^	113.2
Shahdad et al. [35]	Prospective clinical trial	NR	Premolar (13) molar (1)	Maxilla (4) mandible (10)	Mesial (12) distal (2)	No tooth preparation	Retainer wing with partial or complete occlusal coverage	Y‐TZP with veneering of IPS e.max Ceram layering	Vital	Resin adhesive^d^	36.2
Yazigi and Kern [34]	Retrospective clinical study	University	Premolar (18) molar (3)	Maxilla (10) mandible (11)	NR	Minimal preparation within enamel	Retainer wing with occlusal rest	3Y‐TZP with some premolars were veneered with feldspathic ceramic	Vital	Phosphate containing adhesive system^e^	53 ± 39
Lam et al. [38]	Prospective clinical trial	University	Premolar (31) molar (9)	Maxilla (26) mandible (14)	NR	Minimal preparation within enamel	D‐shaped retainer with occlusal bar	Y‐TZP	Vital	Resin adhesive^a^	49.4
Kasem et al. [41]	Prospective randomized clinical trial	University	Premolar (40) molar (0)	Maxilla (0) mandible (40)	Mesial (40) distal (0)	Minimally invasive inlay‐retained preparation confined mostly to enamel (proximal box in dentin)	Inlay retainer with lingual or occlusal coverage	Monolithic zirconia (*n* = 20) or fiber‐reinforced composite (*n* = 20)	Vital; 1 abutment endodontically treated during follow‐up	Resin adhesive^a^	36.0

Abbreviations: C‐FDP, cantilever fixed dental prosthesis; NR, not reported; Y‐TZP, yttria‐stabilized tetragonal zirconia polycrystalline.

^a^Panavia, Kuraray co., Osaka, Japan.

^b^Harvard dental.

^c^Ivoclar Vivadent (Tetric Flow, Tetric Ceram, and Variolink), 3M ESPE (Rely‐X), KURARAY (Panavia F), Optident (HFO).

^d^Multilink Automix, Ivoclar Vivadent.

^e^Manufacturer’s details have not been mentioned.

^f^Lithium disilicate or leucite‐reinforced glass ceramic, leucite‐reinforced.

Considerable heterogeneity was observed in the retainer preparation and configuration, framework material, and cementation protocol. Tooth preparation strategies ranged from no‐preparation and enamel‐confined designs to defect‐oriented, full‐crown, D‐shaped, and minimally invasive inlay‐retained designs [22, 24, 34–41]. Cast metal, porcelain fused to metal (PFM), zirconia, glass‐ceramic, alumina, and fiber‐reinforced composite frameworks were reported [22, 24, 34–41]. Adhesive cementation was used for 373 prostheses [22, 24, 34–36, 38], whereas zinc phosphate cementation was used for seven full‐crown retainers [37]. The principal clinical and prosthetic characteristics are summarized in Table 1 and Figure 2.

**Figure 2:**
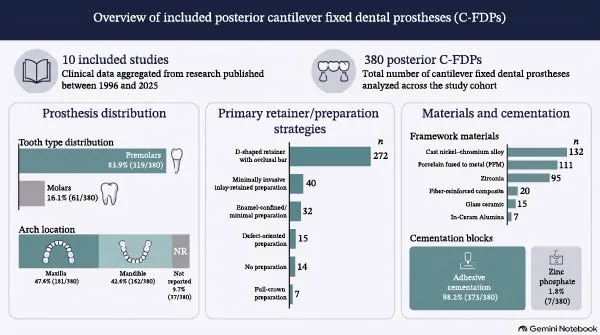
Visual summary of the clinical and prosthetic characteristics of the posterior cantilever fixed dental prostheses included in the review. Figure generated using NotebookLM (Google) [33]. Arch location was reported for 343 prostheses. Data were derived from Table 1. C‐DDP, cantilever fixed dental prosthesis; NR, not reported.

### 3.3. Quality Assessment

The quality of the included studies was appraised by the JBI assessment tool for case series studies [32]. Seven studies were classified as high‐quality studies, while the remaining three were categorized as medium‐quality studies (Supporting Information: Appendix C and Figure 3).

**Figure 3:**
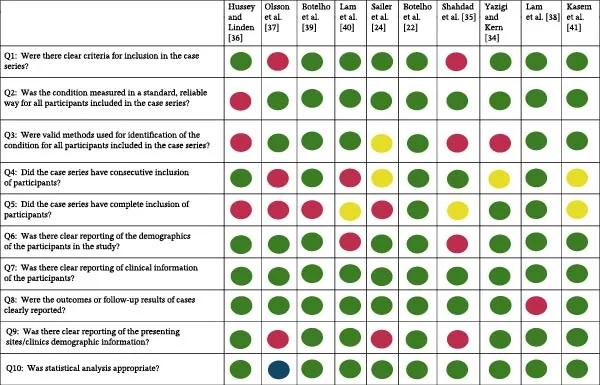
Color‐coded quality assessment of the included studies: “yes” is presented with green, “no” is presented with red, “unclear” is presented with yellow, and “not applicable” is presented with blue circles.

In addition to the JBI assessments, potential sources of bias were identified across the included studies. Common methodological limitations involved small sample sizes, limited reporting of patient selection criteria, and a lack of consecutive recruitment. Furthermore, heterogeneity was noted in several domains, including cantilever design, restorative materials, preparation strategies, cementation protocols, and outcome reporting. These variations may limit comparability across studies and may introduce selection, performance, and reporting bias [42].

### 3.4. Outcome Measures

#### 3.4.1. Prostheses Survival

Among the 10 included studies, two did not report on the number of failures of posterior C‐FDPs [37] and, therefore, were excluded from the quantitative analysis. Overall, 28 failures occurred in a total of 351 C‐FDPs (8.0%), resulting in a prosthesis survival rate of 95.57% and estimated survival rates of above 93.45% at 3 and 5 years (Table 2).

**Table 2:** Annual failure rates and survival of the prostheses.

Author (year of publication)	No. of total prostheses	Mean follow‐up time (year)	Number of failures	Total prostheses exposure time (year)	Estimated annual failure rate (per 100 prostheses years)	Estimated survival rate (in percent)
At least 3‐year follow‐up						
Hussey and Linden (1996) [36]	11	3.06	0	33.66	0	100
Botelho et al. (2006) [39]	110	4.30	9	473.92	1.90	94.46
Shahdad et al. (2018) [35]	14	3.01	2	42.14	4.74	85.78
Yazigi and Kern (2022) [34]	21	4.41	0	92.61	0	100
Lam et al. (2024) [38]	40	4.11	2	164.4	1.21	96.37
Kasem et al. (2025) [41]	40	3	1	120.00	0.83	97.53
Sum	236	3.64	14	926.73	1.51	95.57
Random‐effects summary incidence rate (95% CI)	—	—	—	—	1.82 (1.10, 3.02)	—
At least 5‐year follow‐up						
Sailer et al. (2013) [24]	15	6	0	90	0	100
Botelho et al. (2014) [22]	100	9.43	14	943	1.48	92.6
Sum	115	7.71	14	1033	1.36	93.45
Random‐effects summary incidence rate (95% CI)	—	—	—	—	1.44 (0.86, 2.40)	—
Overall results	351	4.67	28	1959.73	1.43	—
Overall random‐effects summary incidence rate (95% CI)	—	—	—	—	1.62 (1.13, 2.32)	—

*Note:* NR, Lam et al. [38] reported failures only for the combined 39‐case cRBB cohort; the posterior‐only (22‐case) failure count could not be isolated from the published data.

##### 3.4.1.1. Premolar Versus Molar

The survival outcomes based on the site of the missing tooth, premolar versus molar, are presented in Table 3. Four studies [35, 37, 38, 40] were excluded for this comparison, as they did not report on the site of the failed prostheses. The remaining six studies [22, 24, 34, 36, 39, 41] with 256 premolar replacing prostheses had a mean follow‐up time between 3.00–9.43 years (mean 5.03 years) and a total of 19 failed prostheses. The estimated annual failure rate was 1.28%, translated into 3‐ and 5‐year estimated survival rates of 96.24% and 93.82%, respectively. For molar replacing C‐FDPs, 5 out of the total 41 prostheses failed, giving an estimated annual failure rate of 1.89% and estimated 3‐ and 5‐year survival rates of 94.49% and 90.98%, respectively. The meta‐analysis did not reveal any significant differences in the survival rate between premolar and molar‐replacing prostheses (Figure 4A).

**Figure 4:**
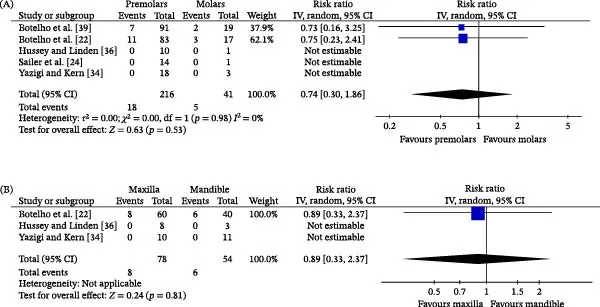
Annual failure rates of (A) the premolar or molar replacing prostheses, and (B) the maxillary and mandibular prostheses.

**Table 3:** Annual failure and survival rates of the C‐FDPs according to location in the mouth.

Author (year of publication)	No. of prostheses	Mean follow‐up time (year)	Number of failures	Total prosthesis exposure time (years)	Estimated annual failure rate (per 100 prostheses years)	Estimated 3‐year survival rate (%)	Estimated 5‐year survival rate (%)
Premolar							
Hussey and Linden (1996) [36]	10	3.06	0	30.6	0	100	100
Botelho et al. (2006) [39]	91	4.31	7	392.06	1.79	94.78	91.46
Sailer et al. (2013) [24]	14	6	0	84	0	100	100
Botelho et al. (2014) [22]	83	9.43	11	782.69	1.40	95.8	93
Yazigi and Kern (2022) [34]	18	4.41	0	79.38	0	100	100
Kasem et al. (2025) [41]	40	3.00	1	120.00	0.83	97.53	95.92
Sum	256	5.03	19	1488.73	1.28	96.24	93.82
Random‐effects summary incidence rate (95% CI)	—	—	—	—	1.43 (0.93, 2.21)	—	—
Molar							
Hussey and Linden (1996) [36]	1	3.06	0	3.06	0	100	100
Botelho et al. (2006) [39]	19	4.31	2	81.86	2.44	92.93	88.50
Sailer et al. (2013) [24]	1	6	0	6	0	100	100
Botelho et al. (2014) [22]	17	9.43	3	160.31	1.87	94.39	90.65
Yazigi and Kern (2022) [34]	3	4.41	0	13.23	0	100	100
Sum	41	5.44	5	264.46	1.89	94.49	90.98
Random‐effects summary incidence rate (95% CI)	—	—	—	—	2.84 (1.32, 6.13)	—	—
Risk ratio: 0.74 (0.30, 1.86)							
Maxilla							
Hussey and Linden (1996) [36]	8	3.06	0	24.48	0	100	100
Botelho et al. (2006) [39]^a^	68	4.31	NR	NR	NR	NR	NR
Botelho et al. (2014) [22]	60	9.43	8	565.8	1.41	95.77	92.95
Yazigi and Kern (2022) [34]	10	4.41	0	44.1	0	100	100
Sum	146	5.30	8	634.38	1.26	96.29	93.89
Random‐effects summary incidence rate (95% CI)	—	—	—	—	1.43 (0.74, 2.74)	—	—
Mandible							
Hussey and Linden (1996) [36]	3	3.06	0	9.18	0	100	100
Botelho et al. (2006) [39]^a^	42	4.31	NR	NR	NR	NR	NR
Botelho et al. (2014) [22]	40	9.43	6	377.2	1.59	95.23	92.05
Yazigi and Kern (2022) [34]	11	4.41	0	48.51	0	100	100
Kasem et al. (2025) [41]	40	3.00	1	120.00	0.83	97.53	95.92
Sum	136	4.84	7	554.89	1.26	96.29	93.89
Random‐effects summary incidence rate (95% CI)	—	—	—	—	1.54 (0.77, 3.08)	—	—
Risk ratio 0.89 (0.33, 2.37)							

Abbreviation: NR, not reported.

^a^Botelho et al. [39] was excluded from the calculation of pooled annual failure rates, estimated survival rates, and summary incidence‐rate estimates for the maxillary and mandibular subgroups because arch‐specific failure data for the posterior prostheses could not be isolated from the published data.

##### 3.4.1.2. Maxillary Versus Mandibular

The survival outcomes based on jaw location, maxillary *versus* mandibular C‐FDPs, are presented in Table 3. Four studies were included [22, 34, 36, 39], with a total of 146 maxillary prostheses and eight failures that occurred within a follow‐up time between 3.06 and 9.43 years (mean 5.30 years). The estimated annual failure rate was calculated at 1.26%, corresponding to 3‐ and 5‐year estimated survival rates of 96.29% and 93.89%, respectively [22, 34, 36]. Mandibular C‐FDPs, on the other hand, included five studies with 136 prostheses and seven failures within 3.00–9.43 years (mean 4.84 years) [22, 34, 36, 39, 41]. This resulted in an annual failure rate of 1.26% and 3‐ and 5‐year estimated survival rates of 96.29% and 93.89%, respectively. The meta‐analysis did not reveal any significant statistical differences in the survival rate between the maxillary and mandibular C‐FDPs (Figure 4B).

##### 3.4.1.3. Mesial‐Versus Distal‐Cantilever

Two studies reported on the survival outcome in relation to the pontic site of the C‐FDPs (mesial or distal cantilever) [24, 36]. Of the total 26 C‐FDPs reported in both studies and followed for a period between 3.06 and 6 years, 21 were mesial cantilever and 5 were distal cantilever prostheses. No failure of any of the prostheses was reported in both studies, resulting in an estimated survival rate of 100% over a 3–5‐year period.

#### 3.4.2. Biological Complications

The biological complications and rates reported in the included studies are presented in Table 4. In three studies [24, 34, 36], no complications were reported, resulting in a 0% incidence rate. In one study [34], abutment teeth developed caries on tooth surfaces not related to the retainer, which was treated without compromising the prostheses. The study by Lam et al. [38] reported on the formation of a deep periodontal pocket in one abutment tooth. The study by Kasem et al. [41] reported one biological complication among posterior prostheses, in which an abutment tooth required endodontic treatment after 12 months. The remaining five studies [22, 35, 37, 39, 40] did not specifically report on the biological outcome or complications of the C‐FDPs attributable to posterior premolar‐ or molar‐replacing prostheses.

**Table 4:** Complications, failure, and survival rate of the C‐FDPs.

Author (year of publication)	Failure rate, *n* (%)	Mechanical complication rate, *n* (%)	Biological complication rate, *n* (%)	Survival rate (%)	No. of prostheses
Hussey and Linden (1996) [36]	0 (0)	0 (0)	0 (0)	100	11
Olsson et al. (2003) [37]	NR^1^	NR	NR	NR	7
Botelho et al. (2006) [39]	9 (8.2)	NR	NR	91.8	110
Lam et al. (2013) [40]	NR^2^	NR	NR	NR	22
Sailer et al. (2013) [24]	0 (0)	1 (6.6).	0 (0)	100	15
Botelho et al. (2014) [22]	14 (14)	NR	NR	86	100
Shahdad et al. (2018) [35]	2 (14.2)	NR	NR	85.8	14
Yazigi and Kern (2022) [34]	0 (0)	0 (0)	0 (0)	100	21
Lam et al. (2024) [38]	2 (5)	0 (0)	1 (2.5)	88.1	40
Kasem et al. (2025) [41]	1 (2.5)	2 (5)	1 (2.5)	100	40

*Note:* NR^1^, data specific to the C‐FDPs was not reported. NR^2^, data specific to posterior C‐FDPs was not reported.

With regard to the vitality status of abutment teeth, five studies used C‐FDPs over vital abutment teeth with no reports of any loss of vitality over the observation period [24, 34–36, 38]. On the other hand, one study [22] reported loss of vitality in some of the abutment teeth for which endodontic treatment was required but without specifying whether these were abutments of premolar‐ or molar‐replacing prostheses. The study by Kasem et al. [41] similarly reported the loss of vitality in one abutment tooth requiring endodontic treatment. In another study [37], the vitality status of the abutment teeth was not reported.

#### 3.4.3. Mechanical Complications

With regard to mechanical complications, no events were encountered in three studies [34, 36, 38]. While minor ceramic chipping not requiring intervention was reported in another study [24]. The study by Kasem et al. [41] reported two mechanical complications among posterior prostheses: one debonding of a zirconia lingual‐coverage retainer after 10 months, which was successfully rebonded, and one connector fracture of a zirconia occlusal‐coverage retainer after 30 months, which required replacement of the prosthesis. In the remaining five studies [22, 35, 37, 39, 40], it was not possible to extract the frequency of mechanical complications of the C‐FDPs attributable specifically to posterior prostheses as the data was pooled to the entire study sample.

### 3.5. Meta‐Analysis

Quantitative analysis was possible in eight of the included studies [22, 24, 34–36, 38, 39, 41]. The summary annual failure rate per 100 years of the prostheses, regardless of the variables, was calculated to be 1.62 (1.13–2.32) with a 95% confidence interval.

Considering premolar and molar C‐FDPs, Figure 4A depicts the meta‐analysis performed for 7 and 2 failure events observed in premolar and molar C‐FDPs.

Considering maxillary and mandibular C‐FDPs, Figure 4B presents the meta‐analysis conducted for 8 and 6 failure events that occurred in 78 and 54 maxillary and mandibular prostheses, respectively.

## 4. Discussion

This systematic review and meta‐analysis presented the survival and complication outcomes of posterior tooth‐supported C‐FDPs over an observation time of at least 3 years. Cantilevered prostheses become particularly significant when other replacement options—such as implant‐supported prostheses or removable prostheses—are not possible due to various patient‐ or site‐related factors. From a clinical perspective, it is also relevant to consider alternative strategies for single‐tooth replacement. Systematic reviews report that implant‐supported single crowns demonstrate excellent longevity, with estimated 5‐year survival rates of ~94%–96% [43]. Conventional three‐unit tooth‐supported FDPs also show favorable outcomes, with reported 5‐year survival around 93%–94% [43]. These data indicate that implant‐supported single crowns, conventional FDPs, and C‐FDPs could all be considered as valid treatment options for replacement of a single missing posterior tooth, with the choice depending on patient‐ and site‐specific feasibility. The review revealed that different types of C‐FDPs could be considered to replace a single missing tooth in the posterior area, with overall survival rates above 90% over 3–5 years of follow‐up. However, only 10 studies ultimately fulfilled the inclusion criteria, and most were observational in design with modest sample sizes. This limited evidence base substantially restricts the strength of the conclusions that can be drawn and reduces the generalizability of the findings to broader patient populations. The review also demonstrated comparable survival rates for premolar and molar replacing C‐FDPs at 3 and 5 years. While long‐term outcomes of tooth‐supported C‐FDPs are rarely reported, their performance seems to be influenced by their anterior or posterior location. For example, Thoma et al. [44] have shown higher incidence of debonding and lower survival rates for posterior compared to anterior tooth‐supported C‐FDPs. The authors, however, did not provide separate outcome analysis for premolar and molar prostheses, which could be related to the fact that only one molar C‐FDP was included, while the remaining were all premolar C‐FDPs.

In the present systematic review, the survival rates of maxillary and mandibular C‐FDPs were comparable at both time intervals. By contrast, the study of Thoma et al. [44] showed that C‐FDP prostheses replacing missing teeth in the maxilla have higher survival rates than those in the mandible. Similarly, in the present review, mesial versus distal C‐FDPs demonstrated a 100% survival rate at 3 and 5 years. Unfortunately, these findings cannot be compared or contrasted with others due to the lack of similar studies. Nevertheless, the findings of the present review need to be interpreted cautiously due to potential bias related to the limited number of studies and the small number of prostheses included. Moreover, no sensitivity analysis was undertaken to explore whether the exclusion of medium‐quality studies would alter the pooled estimates, and therefore, the potential influence of study quality on calculated failure rates could not be assessed within the scope of this review. While this may limit the assessment of the robustness of the pooled estimates, the consistency of findings across included studies suggests that the overall conclusions are unlikely to be substantially altered. Considering abutment tooth preparation and retainer design of C‐FDPs, the literature is also scarce in reporting, with two systematic reviews on RBFDPs available [23, 45]. In these reviews, however, no comparisons have been made between the different designs of the C‐FDPs, indicating a current lack of information on this aspect. In the present review, however, minimally invasive preparation limited to enamel with a wraparound retainer and cingulum rests as well as cavity‐oriented preparations showed a 100% estimated survival rate. The D‐shaped retaining units introduced in the studies by Botelho et al. [22] showed the second‐highest estimated survival rate at 3 and 5 years [38]. In contrast, the lowest survival rate was reported by Shahdad et al. [35], whose design involved a lingual/palatal retaining wing with no tooth preparation. This finding, however, should not be a justification for carrying extensive tooth preparation in order to improve the survival outcome of the C‐FDPs.

With regards to the material used in the fabrication of the C‐FDPs, all ceramic prostheses using lithium disilicate and leucite‐reinforced glass ceramic showed the highest survival rate at both time points, followed by PFM and Y‐TZP. To the best knowledge of the authors, there is no literature that has compared different materials of C‐FDPs exclusively in posterior sites. The aforementioned review of Thoma et al. [44] while describing only resin‐bonded C‐FDPs of different materials, yet the survival analysis included both anterior and posterior C‐FDPs. The highest 5‐year survival rate was reported for C‐FDPs made up of zirconia frameworks (100%), followed by reinforced glass ceramic frameworks (95.3%), and PFM C‐FDPs with 91.3%.

The impact of the type of cement on the outcome of the C‐FDPs could not be definitely assessed in this review. The studies showed that the estimated survival rates for different phosphate‐containing resin cements at 3 years and 5 years were significantly higher compared to the other cement. While this could be an indication of the superiority of phosphate‐containing resin cement, the higher number of prostheses and longer follow‐up times were noted for these studies compared to the study using other cement, which needs to be considered.

Regarding the biological and mechanical complications of the C‐FDPs, the results seem to be inconclusive, with five of the 10 included studies not reporting on these outcomes. In the remaining five studies, however, there was one biological complication [38], while one event of ceramic chipping resulting was reported in another study [24]. In addition, Kasem et al. [41] reported two mechanical complications (one debonding that was successfully rebonded and one connector fracture requiring replacement) and one biological complication (an abutment requiring endodontic treatment) among their 40 prostheses. Compared to the study of Thoma et al. [44] on resin‐bonded C‐FDPs, secondary caries, periodontitis, and abutment tooth fracture were reported at an annual rate of 0.34%, 0.17%, and 0.03%, respectively. The mechanical complications reported, on the other hand, were debonding as the most common complication with an annual rate of up to 12.8%, while ceramic chipping was estimated at an annual rate of 0.84%.

The present systematic review has several limitations, related mainly to the small number of studies and the number of C‐FDPs included, that need to be acknowledged. The lack of well‐designed studies with larger sample sizes evaluating premolar or molar cantilevered prostheses is one major reason, coupled with the strict inclusion criteria of a minimum of 3 year follow‐up of prostheses in function. In addition, many potential studies were excluded due to the lack of details of the specific design, material, or location of the missing tooth in the dental arch. The lack of randomized controlled studies did not allow for objective assessment of confounding variables and subsequent bias in the analysis of the results described in this systematic review. Furthermore, most of the included studies were observational in nature, and the number of reported failure events was low. Consequently, several meta‐analytic estimates were associated with wide confidence intervals, indicating substantial statistical imprecision and limiting the reliability and generalizability of the pooled findings. Although quantitative synthesis was undertaken to summarize the limited available comparative evidence, the resulting estimates should be interpreted as exploratory and should not be considered evidence of equivalence between the compared groups. Several studies reported zero events in both comparison groups, and the small number of studies contributing informative events also precluded a meaningful leave‐one‐study‐out sensitivity analysis as exclusion of individual informative studies would have left insufficient data for a stable pooled estimate. However, the present review provides the best available evidence on the outcome of posterior tooth‐supported C‐FDPs and underscores the need for more well‐designed long‐term clinical trials to validate the findings presented.

## 5. Conclusion

Within the limitations of this review, posterior tooth‐supported C‐FDPs could be considered a valid treatment option for the replacement of a single missing posterior tooth, showing a relatively high 3–5‐year survival rate; however, this should be interpreted cautiously given the limited evidence. Comparable performance was observed between maxillary and mandibular sites, premolar and molar regions, and mesial and distal C‐FDPs. Minimally prepared abutment teeth may show better performance than nonprepared teeth, although the evidence remains limited. Biological and mechanical complications appear minimal; however, outcomes across different C‐FDP designs remain inconclusive due to the small number of included studies. Overall, the review findings should be considered preliminary and interpreted cautiously and contextualized for each clinical case rather than being viewed as definitive evidence. Further well‐designed, long‐term randomized, or prospective studies are required.

## Funding

This study was conducted without financial support from any funding agencies or organizations.

## Disclosure

This review shows that C‐FDP could be considered a valid treatment option for replacing a single posterior unit in the short‐ to mid‐term.

## Conflicts of Interest

The authors declare no conflicts of interest.

## Supporting Information

Additional supporting information can be found online in the Supporting Information section.

## Supporting information


**Supporting Information** Appendix A‐ Search terms used in each electronic database. Appendix B‐ Excluded studies and reasons for exclusion. Appendix C‐ Quality assessment of included studies, using JBI case series quality assessment tool.

## Data Availability

The data that support the findings of this study are available in the Supporting Information of this article.
