# Supplementary material for: Clinical Performance of Posterior Tooth‐Supported Cantilevered Prostheses With a Follow‐Up of ≥ 3 Years: A Systematic Review and Meta‐Analysis
Source: Int J Dent. 2026 Sep 30;2026:9315051. doi: 10.1155/ijod/9315051 (PMC13627887; doi:10.1155/ijod/9315051)
Supplement: Supplementary file 1 [file IJOD-2026-9315051-s001.docx]

**APPENDIX A - Search terms used in each electronic database.**

| Search terms | |
| --- | --- |
| **PubMed / Medline** | **Total results** |
| ((((((((Humans[MeSH Terms]) OR (Adolescent[MeSH Terms])) OR (Adult[MeSH Terms])) OR (Individuals with cantilevered restoration)) OR (Individuals with restoring a premolar tooth)) OR (Individuals with restoring a molar tooth) AND (clinicaltrial[Filter] OR randomizedcontrolledtrial[Filter])) AND (((((((((((Dental Bonding*[MeSH Terms]) OR (Denture, Partial, Fixed[MeSH Terms])) OR (Dental Veneers / standards[MeSH Terms])) OR (Denture, Partial, Fixed* / standards[MeSH Terms])) OR (Dental Prosthesis[MeSH Terms])) OR (Cantilever[MeSH Terms])) OR (Mouth, Edentulous*[MeSH Terms])) OR (Fixed partial prosthesis[MeSH Terms])) OR (Cantilevered restoration)) OR (Cantilever)) OR (Cantilever fixed dental prosthesis) AND (clinicaltrial[Filter] OR randomizedcontrolledtrial[Filter]))) AND (((((((((((((((((Dental Materials*[MeSH Terms]) OR (Dental Porcelain[MeSH Terms])) OR (Zirconium[MeSH Terms])) OR (zirconium oxide[MeSH Terms])) OR (Complications[MeSH Terms])) OR (Metal-ceramics[MeSH Terms])) OR (Dental Materials / chemistry*[MeSH Terms])) OR (Dental Prosthesis Design*[MeSH Terms])) OR (Dental Bonding / methods*[MeSH Terms])) OR (RBFDPs[MeSH Terms])) OR (Fixed dental prostheses[MeSH Terms])) OR (Single retainer[MeSH Terms])) OR (Molar[MeSH Terms])) OR (Premolar[MeSH Terms])) OR (Different materials of cantilevered restorations)) OR (Performance of the restoration at molar)) OR (Performance of the restoration at premolar) AND (clinicaltrial[Filter] OR randomizedcontrolledtrial[Filter]))) AND ((((((Treatment Outcome[MeSH Terms]) OR (Dental Restoration Failure[MeSH Terms])) OR (Survival Analysis[MeSH Terms])) OR (Dental restoration failure[MeSH Terms])) OR (Success)) OR (Survival rates) AND (clinicaltrial[Filter] OR randomizedcontrolledtrial[Filter])) | **2865** |
|  | **Included** |
|  | **9** |
| **Scopus** | **Total results** |
| ( TITLE-ABS-KEY ( human OR adolescent* OR adult OR premolar* OR molar* ) AND TITLE-ABS-KEY ( "dental bonding*" OR "denture" OR "partial" OR "fixed*" OR "dental prosthesis" OR cantilever* OR mouth OR edentulous OR "fixed partial prosthesis" OR "cantilever* restoration" OR cantilever OR "cantilever* fixed dental prosthesis" ) AND TITLE-ABS-KEY ( "dental material*" OR "dental porcelain" OR "porcelain" OR "zirconium" OR "zirconium oxide" OR complications OR "metal-ceramic" OR "metal ceramic" OR "dental material chemistry*" OR "dental prosthesis design*" OR "RBFDP" OR "resin bonded fixed dental prosthesis" OR "fixed dental prosthesis" OR "single retainer" OR "molar" OR "premolar" OR "different materials of cantilevered restorations" OR "performance of restoration at molar" OR " performance of restoration at premolar" ) AND TITLE-ABS-KEY ( "treatment outcome" OR "outcome" OR "restoration failure" OR "survival analysis" OR "dental restoration failure" OR "success" OR "survival" OR "survival rate" OR "success rate" ) ) AND ( LIMIT-TO ( SUBJAREA , "DENT" ) ) AND ( LIMIT-TO ( LANGUAGE , "English" ) | **9123** |
|  | **Included** |
|  | **8** |
| **Embase** | **Total results** |
| 1 tooth arch/ 5382  2 premolar tooth/ or posterior tooth.mp. or molar tooth/ 45959  3 hypodontia/ or missing tooth.mp. 4532  4 human/ or adult/ or individual.mp. 25936679  5 dental prosthesis.mp. or tooth prosthesis/ 30046  6 FDP.mp. 4496  7 denture/ or tooth prosthesis/ or zirconium/ or fixed dental prosthesis.mp. or fixed partial denture/ 72980  8 fixed partial denture/ or FPD.mp. or denture/ 44572  9 partial denture.mp. or denture/ or tooth prosthesis/ or partial denture/ 61806  10 Maryland bridge.mp. or resin-bonded fixed partial denture/ 204  11 dental bridge.mp. or denture/ or tooth prosthesis/ or dental bridge/ or tooth bridge/ 59697  12 cantilever*.mp. or cantilever dental bridge/ or denture/ or tooth prosthesis/ 64368  13 denture/ or dental extension.mp. 41398  14 resin-bonded fixed partial denture/ or tooth prosthesis/ or fixed partial denture/ or RBFDP*.mp. 29882  15 fixed partial denture/ or denture/ or resin-bonded fixed partial denture/ or single retainer.mp. or tooth prosthesis/ 60453  16 reconstruction material.mp. 700  17 dental material.mp. or "biomedical and dental materials"/ or dental material/ or dental alloy/ 36404  18 porcelain tooth/ or dental porcelain/ or porcelain.mp. 9252  19 cement/ or tooth cement/ or cement.mp. or polycarboxylate cement/ or resin cement/ 63314  20 dental cement.mp. or tooth cement/ 9021  21 zirconia.mp. or zirconium oxide/ 11895  22 tooth prosthesis/ or PFM.mp. or denture/ or tooth crown/ 74844  23 denture/ or dental alloy/ or tooth prosthesis/ or tooth crown/ or porcelain fused to metal.mp. or dental porcelain/ 84113  24 ceramic.mp. or ceramics/ 38756  25 alloy/ or ceramics/ or dental porcelain/ or metal-ceramic.mp. 43713  26 dental prosthesis design.mp. or prosthesis design/ 7456  27 tooth crown/ or onlay retain*.mp. or fixed partial denture/ 23383  28 resin crown/ or resin/ or resin-bonded fixed partial denture/ or resin cement/ or resin denture/ or resin.mp. 105745  29 tooth prosthesis/ or distal cantilever*.mp. or denture/ 59531  30 tooth prosthesis/ or denture/ or mesial cantilever*.mp. or fixed partial denture/ 60378  31 short term survival/ or long-term survival/ or median survival time/ or survival/ or failure free survival/ or survival rate/ or survival analysis/ or mean survival time/ or overall survival/ or survival time/ or survival.mp. 2153468  32 survival rate/ or survival rate.mp. 372561  33 failure.mp. 1835867  34 restoration failure.mp. 353  35 prosthesis failure.mp. or prosthesis complication/ 19090  36 treatment outcome.mp. or outcome assessment/ or treatment outcome/ 1766840  37 dental restoration failure.mp. or dental restoration failure/ 128  38 success.mp. 484373  39 success rate.mp. 92185  40 fracture.mp. or fracture/ or tooth fracture/ 430257  41 loss of retention.mp. 568  42 risk/ or risk.mp. 4905545  43 prosthesis loosening/ or loosening.mp. 33098  44 biological complication*.mp. 526  45 prosthetic complications.mp. 475  46 adverse effects.mp. or adverse event/ 325971  47 cement failure.mp. 90  48 caries.mp. or dental caries/ 65929  49 complication/ or complicat*.mp. 3575511  50 performance.mp. or performance/ 2013520  51 technical complications.mp. 2050  52 complications/ or mechanical complications.mp. 363719  53 1 or 2 or 3 or 4 25944177  54 5 or 6 or 7 or 8 or 9 or 10 or 11 or 12 or 13 or 14 or 15 85981  55 16 or 17 or 18 or 19 or 20 or 21 or 22 or 23 or 24 or 25 or 26 or 27 or 28 or 29 or 30 285468  56 31 or 32 or 33 or 34 or 35 or 36 or 37 or 38 or 39 or 40 or 41 or 42 or 43 or 44 or 45 or 46 or 47 or 48 or 49 or 50 or 51 or 52 12870880  57 53 and 54 and 55 and 56 18125  58 limit 57 to (human and English language) 17630 | **17630** |
|  | **Included** |
|  | **10** |

**APPENDIX B -** Excluded studies and reasons for exclusion

| No. | Author and year of publication | Reason for exclusion |
| --- | --- | --- |
| 1 | Dunne 1993 [1] | Mean observation time is not reported. |
| 2 | Palmqvist 1993 [2] | Details have not been reported. |
| 3 | Botelho 2000 [3] | The mean observation period is less than 36 months. |
| 4 | Zalkind 2003 [4] | The design was a double retainer cantilever resin bonded fixed dental prostheses. |
| 5 | Gilmour 1995 [5] | The observation period is less than 36 months. |
| 6 | Djemal 1999 [6] | Details have not been reported. |
| 7 | Botelho 2002 [7] | The outcome of posterior and anterior restorations are pooled. |
| 8 | Chai 2005 [8] | The observation period is less than 36 months. |
| 9 | Ronay 2014 [9] | The observation period is less than 36 months. |
| 10 | Sasse 2014 [10] | The cantilevered prostheses have two abutment teeth. |
| 11 | King 2015 [11] | Mean observation time is not reported. |
| 12 | Botelho 2020 [12] | The observation period is less than 36 months. |
| 13 | Anweigi 2020 [13] | Mean observation time is not reported. |
| 14 | Qiu 2020 [14] | The observation period is less than 36 months. |
| 15 | Candea 2021 [15] | Details have not been reported. |
| 16 | Zitzmann 2021 [16] | Mean observation time is not reported. |
| 17 | Passia 2023 [17] | The observation period is less than 36 months. |
| 18 | Schierz 2023 [18] | The outcome is quality of life and does not match the outcome measures. |
| 19 | Kasem 2023 [19] | The observation period is less than 36 months. |

| **APPENDIX C -** Quality assessment of included studies, using JBI case series quality assessment tool. | | | | | | | | | | | |
| --- | --- | --- | --- | --- | --- | --- | --- | --- | --- | --- | --- |
|  | Q1: Were there clear criteria for inclusion in the case series? | Q2: Was the condition measured in a standard, reliable way for all participants included in the case series? | Q3: Were valid methods used for identification of the condition for all participants included in the case series? | Q4: Did the case series have consecutive inclusion of participants? | Q5: Did the case series have complete inclusion of participants? | Q6: Was there clear reporting of the demographics of the participants in the study? | Q7: Was there clear reporting of clinical information of the participants? | Q8: Were the outcomes or follow-up results of cases clearly reported? | Q9: Was there clear reporting of the presenting site(s)/clinics demographic information? | Q10: Was statistical analysis appropriate? | Overall score |
| Hussey et al. 1996 | Y | N | N | Y | N | Y | Y | Y | Y | Y | 7 |
| Olsson et al. 2003 | N | Y | Y | N | N | Y | Y | Y | N | NA | 5 |
| Botelho et al. 2006 | Y | Y | Y | Y | N | Y | Y | Y | Y | Y | 9 |
| Lam et al. 2013 | Y | Y | Y | N | U | N | Y | Y | Y | Y | 7 |
| Sailer et al. 2013 | Y | Y | U | U | N | Y | Y | Y | N | Y | 6 |
| Botelho et al. 2014 | Y | Y | Y | Y | Y | Y | Y | Y | Y | Y | 10 |
| Shahdad et al. 2018 | N | Y | N | Y | U | N | Y | Y | N | Y | 5 |
| Yazigi et al. 2022 | Y | Y | N | U | Y | Y | Y | Y | Y | Y | 8 |
| Lam et al. 2024 | Y | Y | Y | Y | Y | Y | Y | N | Y | Y | 9 |
| Kasem et al. 2025 | Y | Y | Y | U | U | Y | Y | Y | Y | Y | 8 |

1.	Dunne S, Millar B. A longitudinal study of the clinical performance of resin bonded bridges and splints. Br Dent J. 1993;174(11):405-11. <https://doi.org/10.1038/sj.bdj.4808186>.

2.	Palmqvist S, Swartz B. Artificial crowns and fixed partial dentures 18 to 23 years after placement. Int J Prosthodont. 1993;6(3).

3.	Botelho MG, Nor LC, Kwong HW, Kuen BS. Two-unit cantilevered resin-bonded fixed partial dentures--a retrospective, preliminary clinical investigation. Int J Prosthodont. 2000;13(1):25-8.

4.	Zalkind M, Ever‐Hadani P, Hochman N. Resin‐bonded fixed partial denture retention: a retrospective 13‐year follow‐up. J Oral Rehabil. 2003;30(10):971-7. <https://doi.org/10.1046/j.1365-2842.2003.01165.x>.

5.	Gilmour A, Ali A. Clinical performance of resin-retained fixed partial dentures bonded with a chemically active luting cement. The Journal of Prosthetic Dentistry. 1995;73(6):569-73. <https://doi.org/10.1016/S0022-3913(05)80118-0>.

6.	Djemal S, Setchell D, King P, Wickens J. Long‐term survival characteristics of 832 resin‐retained bridges and splints provided in a post‐graduate teaching hospital between 1978 and 1993. J Oral Rehabil. 1999;26(4):302-20. <https://doi.org/10.1046/j.1365-2842.1999.00374.x>.

7.	Botelho MG, Chan A, Yiu E, Tse E. Longevity of two-unit cantilevered resin-bonded fixed partial dentures. Am J Dent. 2002;15(5):295-9.

8.	Chai J, Chu F, Newsome P, Chow T. Retrospective survival analysis of 3‐unit fixed‐fixed and 2‐unit cantilevered fixed partial dentures. J Oral Rehabil. 2005;32(10):759-65. <https://doi.org/10.1111/j.1365-2842.2005.01495.x>.

9.	Ronay V, Bindl A, Sahrmann P, Schmidlin PR. Retrospective evaluation of CAD/CAM cantilever reconstructions to restore compromised posterior teeth: a preliminary report. Int J Prosthodont. 2014;27(2). <https://doi.org/10.11607/ijp.3514>.

10.	Sasse M, Kern M, Marré B, Walter MH. Clinical performance of cantilevered fixed dental prostheses abutments in the shortened dental arch. J Dent. 2014;42(3):373-6. <https://doi.org/10.1016/j.jdent.2013.12.013>.

11.	King P, Foster L, Yates R, Newcombe RG, Garrett M. Survival characteristics of 771 resin-retained bridges provided at a UK dental teaching hospital. Br Dent J. 2015;218(7):423-8. <https://doi.org/10.1038/sj.bdj.2015.250>.

12.	Botelho MG, Yon MJY, Mak KCK, Lam WYH. A randomised controlled trial of two-unit cantilevered or three-unit fixed-movable resin-bonded fixed partial dentures replacing missing molars. J Dent. 2020;103:103519. 10.1016/j.jdent.2020.103519.

13.	Anweigi L, Azam A, De Mata C, AlMadi E, Alsaleh S, Aldegheishem A. Resin bonded bridges in patients with hypodontia: Clinical performance over a 7 year observation period. The Saudi Dental Journal. 2020;32(5):255-61. <https://doi.org/10.1016/j.sdentj.2019.10.011>.

14.	Qiu H-Y, Wu S-Y, Fu L. Clinical performance of cantilevered resin-bonded fixed partial dentures for single tooth replacement in elderly patients. J Int Med Res. 2020;48(10):0300060520956810. <https://doi.org/10.1177/0300060520956810>.

15.	Cândea A, Goguţă LM, Frandes M, Jivanescu A. Zirconia Minimally Invasive Partial Retainer Fixed Dental Prostheses: Up To Ten Year Follow-Up. The International Journal of Prosthodontics. 2021;34(6):724–32-–32. <https://doi.org/10.11607/ijp.7061>.

16.	Zitzmann NU, von Büren A, Glenz F, Rohr N, Joda T, Zaugg LK. Clinical outcome of metal-and all-ceramic resin-bonded fixed dental prostheses. J Prosthodontic Res. 2021;65(2):243-8. <https://doi.org/10.2186/jpr.JPR_D_20_00014>.

17.	Passia N, Chaar MS, Kern M. Clinical outcome of posterior cantilever resin-bonded fixed dental prostheses using two different luting agents. J Prosthodont Res. 2023;67(2):161-3. 10.2186/jpr.JPR_D_22_00033.

18.	Schierz O, Lee CH, John MT, Rauch A, Reissmann DR, Kohal R, et al. HOW TO IDENTIFY SUBGROUPS IN LONGITUDINAL CLINICAL DATA: TREATMENT RESPONSE PATTERNS IN PATIENTS WITH A SHORTENED DENTAL ARCH. J Evid Based Dent Pract. 2023;23(1s):101794. 10.1016/j.jebdp.2022.101794.

19.	Kasem AT, Tribst JPM, Abo-Madina M, Al-Zordk W. Evaluation of different designs for posterior cantilever zirconia inlay-retained fixed dental prostheses in missing tooth replacement: Stage one results with 18-month follow-up assessment. J Dent. 2023;137:104688. 10.1016/j.jdent.2023.104688.
